# Phylogeography of Crimean Congo Hemorrhagic Fever Virus

**DOI:** 10.1371/journal.pone.0166744

**Published:** 2016-11-23

**Authors:** Alexander N. Lukashev, Alexander S. Klimentov, Svetlana E. Smirnova, Tamara K. Dzagurova, Jan Felix Drexler, Anatoly P. Gmyl

**Affiliations:** 1 Chumakov Institute of Poliomyelitis and Viral Encephalitides, Moscow, Russia; 2 Institute of Molecular Medicine, Sechenov First Moscow State Medical University, Moscow, Russia; 3 RUDN University, Moscow, Russia; 4 D.I. Ivanovsky Institute of Virology of N.F. Gamaleya Center of Epidemiology and Microbiology, Moscow, Russia; 5 Institute of Virology, University of Bonn Medical Centre, Bonn, Germany; 6 German Centre for Infection Research (DZIF), Bonn-Cologne, Germany; Division of Clinical Research, UNITED STATES

## Abstract

Crimean Congo hemorrhagic fever virus (CCHFV) is one of the most severe viral zoonozes. It is prevalent throughout Africa, Asia and southern Europe. Limited availability of sequence data has hindered phylogeographic studies. The complete genomic sequence of all three segments of 14 Crimean Congo hemorrhagic fever virus strains isolated from 1958–2000 in Russia, Central Asia and Africa was identified. Each genomic segment was independently subjected to continuous Bayesian phylogeographic analysis. The origin of each genomic segment was traced to Africa about 1,000–5,000 years ago. The virus was first introduced to South and Central Asia in the Middle Ages, and then spread to China, India and Russia. Reverse transfers of genomic segments from Asia to Africa were also observed. The European CCHFV genotype V was introduced to Europe via the Astrakhan region in South Russia 280–400 years ago and subsequently gradually spread westward in Russia, to Turkey and the Balkans less than 150 years ago. Only a few recombination events could be suggested in S and L genomic segments, while segment reassortment was very common. The median height of a non-reassortant phylogenetic tree node was 68–156 years. There were reassortment events within the European CCHFV lineage, but not with viruses from other locations. Therefore, CCHFV in Europe is a recently emerged zoonosis that represents a spillover from the global gene pool.

## Introduction

Crimean-Congo hemorrhagic fever (CCHF) is a tick-borne zoonosis that is prevalent in Africa, Asia and Europe. Case-fatality rates range from 5 to 30% [1]. CCHFV virus belongs to the genus *Nairovirus*, family *Bunyaviridae*. The negative-sense RNA genome has three segments. The small (S) segment is about 1.7 Kb long and encodes a nucleoprotein. The medium (M) segment is approximately 5.3 Kb long and encodes a single ORF that is cleaved into two envelope glycoproteins and a hypervariable protein of ca. 250 amino acids known as a mucin-like domain. The large (L) segment is approximately 12.1 Kb long and encodes a single protein that contains a polymerase domain [2]. All segments contain short 5’ and 3’ untranslated regions of 55–170 bases.

CCHFV has several distinct lineages, also termed genotypes (Gt), which have their preferential prevalence regions [3, 4]. Due to segment reassortment [5], there are differences in the lineage pattern in different genome segments. Five to seven genotypes are traditionally designated with Roman numerals. An alternative designation of the same lineages refers to the preferential geographic location [6]. The most ancient lineages (branching close to the tree root in phylogenetic reconstructions) are generally found in Africa, with the exception of strain Ap92, which was isolated in Greece. Most of the European isolates belong to GtV and are much more conserved than CCHFV circulating elsewhere [3, 7, 8]. Strains related to Ap92 (GtVI) are also occasionally found in Europe [7].

CCHFV is relatively poorly represented in sequence databases, partially due to the high biosafety level required to investigate the pathogen. Most of the nucleotide sequences deposited in Genbank correspond to a partial S segment sequence. There are less than 100 complete S segment sequences. There were 39 viruses with complete genomic sequence available for all three genome segments prior to this study. We identified the complete coding sequence of 14 genomes of CCHFV strains that were isolated from 1958–2012 in Russia, Central Asia and Africa and represent several of the genetic lineages.

Phylogeographic studies of CCHFV have been hindered not only by a limited sample, but also by an uneven representation of geographic regions among sequenced viruses. The sample coverage for Europe is better than for Asia and much better than for Africa. Discrete phylogeography may be more robust in this case, and indeed such study based on a partial S segment sequence was published recently [8]. On the other hand, discrete phylogeography relies on arbitrary assignment of location categories, which may be subjective. Continuous phylogeography is less subjective, although certain bias is inevitable because only approximate sampling coordinates were available for most strains. We did a continuous phylogeography analysis independently for three complete genome segments to provide an alternative image of CCHFV evolution.

## Materials and Methods

The viruses were obtained from the collection of the Chumakov Institute of Poliomyelitis and Viral Encephalitides, Moscow, Russia (Table 1). Viruses were stored as lyophilized brain suspensions. RNA was extracted by Trizol reagent (Invitrogen) from mouse brain suspensions. Reverse transcription was carried out using random hexamer primers and Maxima reverse transcriptase (Thermo Scientific). PCR primers were designed to amplify ca. 1,000 nucleotide (nt) fragments of the CCHFV genome overlapping by about 100 nt (S1 Table). Primer specificity was aimed to allow amplification of all known virus genotypes. PCR was carried out using Platinum Taq (Invitrogen) with 2 mM MgCl_2_ and the following cycle: 95°C– 5 min; 10 touchdown cycles (95°C 15”– 65°C to 55°C 20”– 72°C 1 min); 40 amplification cycles (95°C 15”– 55°C 20”– 72°C 1 min); 72°C– 5 min. PCR products were sequenced in both directions by Sanger sequencing. Genomic termini were not sequenced.

**Table 1:** CCHFV strains used in this work and their assignment to virus lineages [3].

Strain	Isolation date	Sampling Location	S segment lineage	M segment lineage	L segment lineage	Genbank No.
Nakiwogo	1958	Uganda	II	I	II	KX013483—KX013485
IbAn 7620	1965	Nigeria	IV	III	III	KX013450—KX013452
Hodzha	1967	Uzbekistan	IV	III	IV	KX013447—KX013449
Saf	1968	Rostov-on-Don, Russia	V	V	V	KX013486—KX013488
Min	1968	Rostov-on-Don, Russia	V	V	V	KX013480—KX013482
Mamon	1968	Rostov-on-Don, Russia	V	V	V	KX013477—KX013479
Gaib	1969	Tajikistan	IV	III	IV	KX013444—KX013446
К128–76	1971	Kazakhstan	IV	III	IV	KX013453—KX013455
К168–125	1973	Turkmenistan	IV	III	IV	KX013459—KX013461
К229–243	1984	Astrakhan, Russia	V	V	V	KX013465—KX013467
К229–194	1989	Astrakhan, Russia	V	V	V	KX013462—KX013464
К315–14	2000	Stavropol Region, Russia	V	V	V	KX013468—KX013470
К323–27	2000	Stavropol Region, Russia	V	V	V	KX013471—KX013473
К340–6	2000	Stavropol Region, Russia	V	V	V	KX013474—KX013476

The resulting sequences were assembled using the SeqMan module of Lasergene package (DNAStar, Inc.). Full or near-full segment CCHFV sequences were extracted from Genbank. Information on date and location of isolation was derived from Genbank records, original papers and previous studies [9]. Detailed sampling locations were available for strains isolated in China, Russia, Iran and India. When a precise sampling location was not known, it was approximated to the country’s location according to the Google country location list (https://developers.google.com/public-data/docs/canonical/countries_csv) or to the endemic Xinjiang region for Chinese isolates. Geographic coordinates used for calculations are available in S2 Table. Sequences that shared over 99.7% identity or did not have a known isolation date/location were omitted.

Phylogenetic analysis was done using the Beast 1.7.5 package [10]. Complete ORF sequences of S and L segments were used for analysis. In the M segment, only sequences encoding glycoproteins were included, while the hypervariable mucin-like domain was excluded. Although inclusion of this domain did not significantly affect the substitution rates in previous studies [9, 11] and in our preliminary runs, its presence destabilized the calculations, resulting in much lower ESS values. The final datasets were thus 1,446, 4,335 and 1,1835 nt long and included 79, 68 and 54 sequences for S, M and L segments, correspondingly. Strain Ap92 isolated in Greece is a highly divergent CCHFV [12]. It was excluded after preliminary analysis to avoid dichotomy of the phylogeny. Continuous phylogeographic calculations were done as previously described [13] using recommended parameters (http://beast.bio.ed.ac.uk/Continuous-phylogeographic-analysis). Computations were run for 60 million (S segment) or 100 million (M and L segments) generations, and trees were sampled every 10,000 generations. ESS values above 200 were achieved for all parameters. Trees were annotated with a 10% burn-in by Tree Annotator provided in the Beast package. Phylogeography was visualized in Google Earth using SPREAD [14]. KML files are available as S1–S3 Files.

Reassortment analysis was done on 53 CCHFV genomes with known complete sequence for all genomic regions. Bayesian phylogenetic trees were constructed as described above with a relaxed lognormal clock and the SRD06 substitution model for 25, 30 and 100 million generations for S, M and L segments, accordingly.

Preliminary recombination analysis was done with the RDP 4.0 package [15]. Bootscan graphs were created with Simplot 3.5.1 [16].

## Results

**Fig 1:**
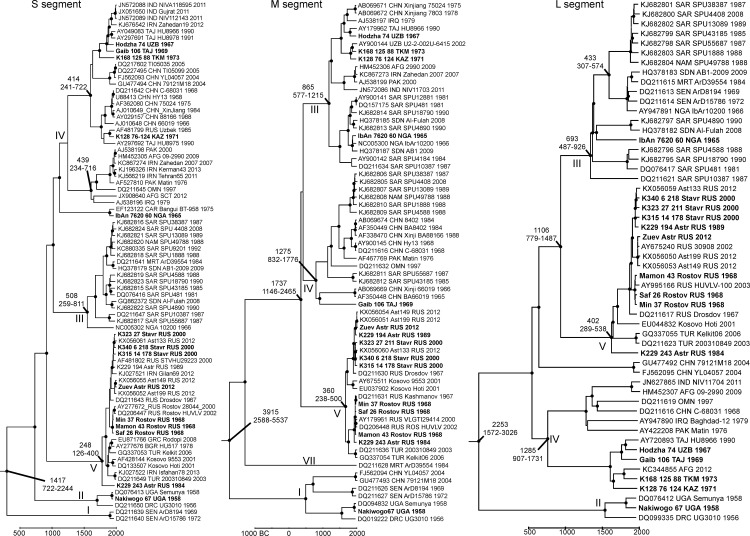
Bayesian phylogenetic trees for complete S, M and L segment sequences. Names of strains sequenced here are indicated in bold. Roman numerals at tree nodes indicate genotypes. The scale bar shows time in years. Nodes with posterior probabilities > 0.95 are marked with black dots. Height and height 95% HPD are indicated for selected nodes.

Complete coding sequences were obtained for 14 CCHFV genomes (Table 1). The genotype of the viruses was identified according to a previous study [3]. Eight viruses belonged to the European genotype V in all genomic segments (Fig 1). Strain Nakiwogo isolated in Uganda in 1958 was very similar to strain Semunya, which was also isolated in Uganda in 1958. However, these viruses differed by 0.4%, 0.8% and 1.9% in S, M and L segments, respectively, and may be regarded as distinct strains. Five strains belonged to the Asian genotype IV in the S segment. Four of them were typical members of this genotype, while isolate IbAn7620 (isolated in Nigeria in 1965) was very similar to strain Bangui BT-958 from the Central African Republic, an outlier within genotype IV. In the M segment, four of these five viruses were genotype III, while strain Gaib isolated in Tajikistan in 1969 was a member of genotype IV and branched close to the genotype’s root. In the L segment, four of the five members of the S segment type IV belonged to the Asian genotype IV; however, strain IbAn7620 belonged to the African genotype III.

Bayesian phylogenetic analysis implied substitution rates of 1.3x10^-4^ substitutions/site/year (ssy), a 95% HPD (high probability density) range [0.62–2.0 x 10^−4^] for the S segment, 1.0 x 10^−4^ [0.65–1.4 x 10^−4^] for the M segment and 0.8 x 10^−4^ [0.6–1.1x10^-4^] for the L segment (Fig 1). These values were in concordance with previously reported rates of 1.09 x 10^−4^, 1.52 x 10^−4^ and 0.58 x 10^−4^ ssy in the S, M and L segments [9] and 1.71 x 10^−4^ [1.25–2.30 x 10^−4^] ssy within GtV [7]. Lower substitution rates reported previously in the L segment were suggested to have resulted from a small sample size [9]. Indeed, a larger sample size in our study might explain slightly higher substitution rates in the S and L segments. In general, substitution rates estimated upon full-segment analysis of different segments and in different studies were very similar. Much higher substitution rates (2.96 x 10^−4^ [1.6–4.7 x 10^−4^] ssy and 1.7–5.7 x 10^−4^ ssy) have been previously reported for partial (ca. 300 or 500 nt) S segment sequences [7, 8]. When we ran the analysis using the same sequences as in the full-segment dataset, but using only the S segment fragment used by Sherifi et al. (2014) the substitution rate was 3.4 x 10^−4^ [1.76–5.51 x 10^−4^] instead of 1.3 x 10^−4^ [0.62–2.0 x 10^−4^] for the full S segment. Therefore, shorter S segment fragments indeed produce higher substitution rates than the complete S segment.

Phylogenies of distinct segments were largely discordant due to reassortment, which is typical for CCHFV [5]. There is no reason for reassortment to affect phylogenetic dating; therefore, reassortant sequences were not excluded. Consistent with previous reports [17], recombination evidence was identified in few sequences (see below). Exclusion of these sequences did not affect the tree topology, substitution rates or other run parameters (data not shown); therefore, they were not omitted from the final runs.

The tree root of all three segments mapped to Central or West Africa. However, 95% HPDs of tree roots were extremely wide: 4°S– 19°N and 20°W– 27°E for S segment; 1°S– 29°N and 21°W– 35°E for M segment; 10°S– 40°N and 11°E– 71°E for L segment; therefore, it is likely that CCHFV originated in Africa; however no more precise conclusions on viral origin could be made. The root age was 1902 [949–3023], 3915 [2588–5537] and 2253 [1572–3026] years for S, M and L segments, respectively. Interestingly, while the lowest substitution rate was predicted for the L segment, the M segment root had a much older age estimate. This may imply that some L and S segment lineages were extinct, or were not sampled yet. The root ages were comparable with those reported previously: 3,138, 3,560 and 7,358 years for the S, M and L segments, respectively [9]. More recent root ages in our study are concordant with higher estimated substitution rates and could be affected by increased sample size. It is likely that further increases of the sample size would result in even more recent root dates.

**Fig 2:**
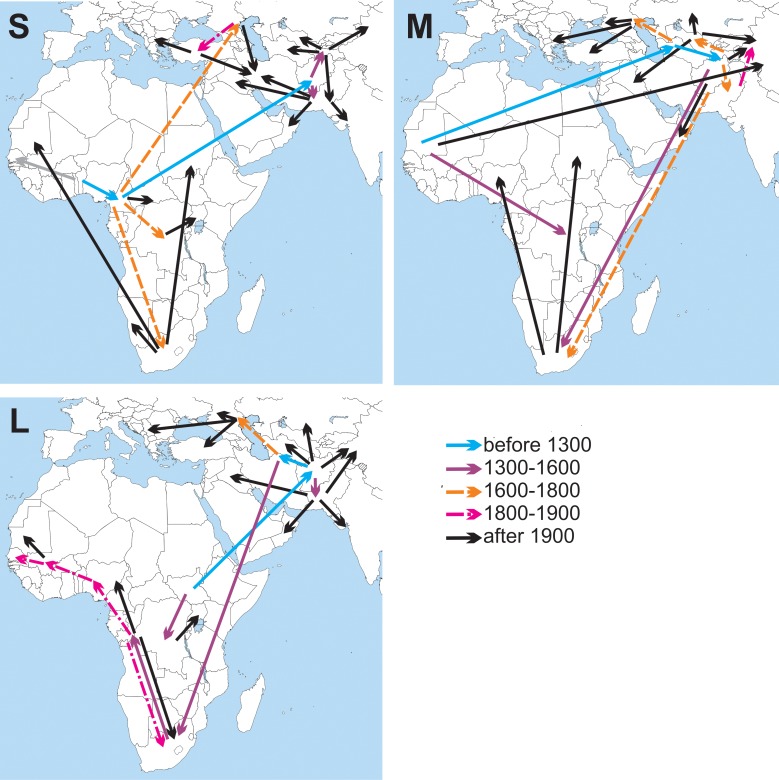
Schematic maps of CCHFV segment spread projected by Bayesian phylogeographic analysis. Panels indicate spread patterns inferred for S, M and L segments. Time of transfers (defined by age of a recipient node) is indicated by line color and style. The draft map was obtained at Wikimedia commons.

The patterns of viral spread suggested by phylogeographic analysis were plotted for each segment (Fig 2). Routes of viral spread in Africa included many long distance jumps and branches with a duration exceeding one thousand years, and were generally discordant in the three genomic segments. Obviously, the current sample of African strains is not sufficient to analyze viral spread in Africa.

The virus was introduced to Central and South Asia in the Middle Ages. The most recent common ancestor (MRCA) of all three segments of Asian CCHFV lineages maps to Middle and Central Asia (Turkmenistan and Afghanistan). The Asian MRCA dates back 1,738 [1,146–2,465] years in the M segment (node posterior probability 1) and 1,509 [1,058–2,31] years in the L segment (node posterior probability 1). Analysis of the S segment phylogeny suggested two independent introductions to South Asia 847 [441–1377] years ago (node posterior probability 0.84) and to South Russia 248 [126–400] years ago (node posterior probability 1). However, this pattern may be an artifact and could be explained by a single ancient introduction and a reverse transfer of virus from Asia to Central Africa. Indeed, long-distance transfers of virus could be suggested for all genome segments according to the topology of the phylogenetic tree, and have been also discussed previously [18, 19].

Complex virus networks were predicted in South Asia, mostly in Afghanistan and Pakistan (not shown of Fig 2). From this ancient focus, the virus was introduced to China, India, Central Asia, South Russia, Iran, Iraq and the Arabian Peninsula. Phylogenetic analysis of three genome segments unanimously points to a single introduction of GtV to Europe. The MRCA of the European GtV (always supported by posterior probability 1) dated back 280 [133–485] years in the S segment, 396 [238–500] years in M and 402 [289–538] years in L segment. This node was dated 1930 [1890–1960] in another study that relied upon partial S segment sequences [7]. Both the use of a full S segment and the inclusion of additionally sequenced viruses could result in a three times older age of this node in our study. Indeed, one of the viruses sequenced in our work (K229-243 Astrakhan RUS 1984) was an outlier within GtV in the S and L segments, which could increase the MRCA age and complement to the signal for mapping the GtV root to Astrakhan.

For all three segments, the predicted introduction point of GtV to Europe was the Astrakhan region including the Volga River Delta. This location is a major virus biodiversity area because many migratory birds make a stop-over or reside there [20]. In addition, this region is geographically closest to Central Asia; therefore, there was an ecological background for both gradual virus spread by the coast of the Caspian Sea and for introduction by migrating birds. The branch leading to GtV has a length of 867, 1,328 and 688 years in the S, M and L segments, so any suggestion on the mechanism of virus introduction to South Russia would be highly speculative.

From the Astrakhan region, GtV spread westward in Russia towards the Black Sea to Rostov and Stavropol regions, to Turkey and to the Balkans. Analysis of S segment phylogeny suggested that the virus spread to the Balkans via Turkey, while the M and L segments implied an independent virus introduction from Astrakhan to Europe and to Turkey. The first hypothesis seems more plausible because the sample of S segment sequences is larger and because it was concordant with the previous estimates [18]. A larger sample of sequences of European viruses is available for a 536-nt fragment of the S segment. Unfortunately, the resolution was relatively low in the partial S segment dataset and did not allow reliably inferring viral spread in Europe on a relatively short time scale [21].

**Fig 3:**
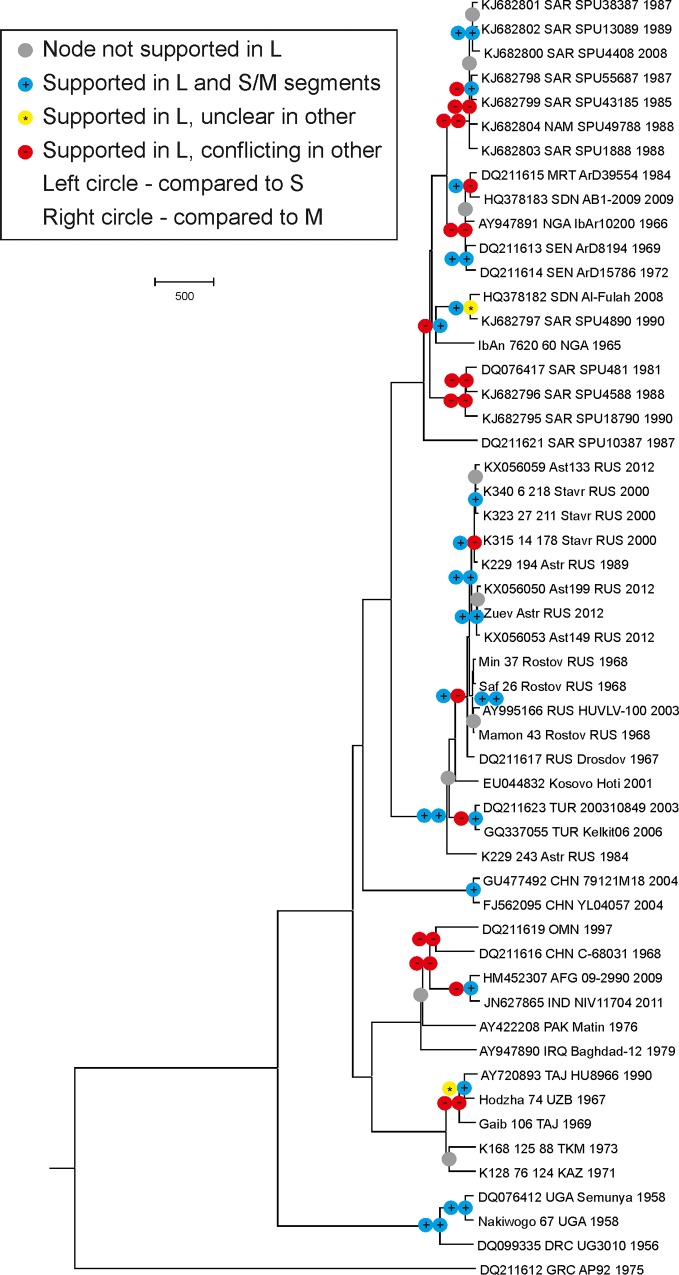
Non-reassortant CCHFV genomes. A Bayesian phylogenetic tree was built for the L segments of 53 CCHFV genomes with available complete coding sequences of all three segments. Poorly supported (posterior probability < 0.95) nodes are indicated in gray. For nodes that were reliably supported in L segment, the conservation of the node in S and M segments is indicated by the left and the right circle, respectively. Tree nodes that were conserved are marked with a blue “+”. Nodes with a reliably supported conflicting phylogeny observed in the S or M segments are marked with a red “-“. Nodes that were not reliably supported in the S or M segments, but were not obviously reassortant, are indicated with a yellow “*”. Nodes that were ancestral to a reassortant node were not analyzed.

The phylogenies of the three segments were largely discordant. Full segments provided very good resolution (robust bootstrap support) even among closely related viruses; therefore, we could analyze reassortment dynamics in detail. For this purpose, a dataset of 53 viruses with complete sequences in all three segments was used. Bayesian phylogenetic analysis revealed only eight tree nodes that were identical and reliably supported (posterior probability values > 0.95) in all three segments (Fig 3). The median age of non-reassortant viruses (distance from a non-reassortant node to strain isolation) was 68, 156 and 77 years in S, M and L segments, respectively. These numbers may be further reduced upon sample increase in the future. There was apparently no limit on the compatibility of segments, because reassortment could involve distantly related viruses that branched close to the tree root. There have been reports on reassortment between the S and L segments [9], but also suggestions of S and L segment co-evolution [22, 23]. In our dataset, there was no evidence that reassortment between the S and L segments was less frequent than that between the L and M segments. One distinct group that was apparently not involved in reassortment included three viruses from DRC and Uganda (GtII in S and L segments). This group branched from other CCHFV lineages over a thousand years ago. Further sampling is required to confirm its involvement in reassortment events. Noteworthy, the genetic distance between the Nakiwogo and Semunya strains was five times higher in the S than in the L segment (see above), which could imply reassortment within the group with unidentified partners. Within the 17 GtV viruses there was evidence of reassortment events (best exemplified by strain K229-243 Astrakhan RUS 1984), but none of them involved introduction of genomic segments from other genotypes.

**Fig 4:**
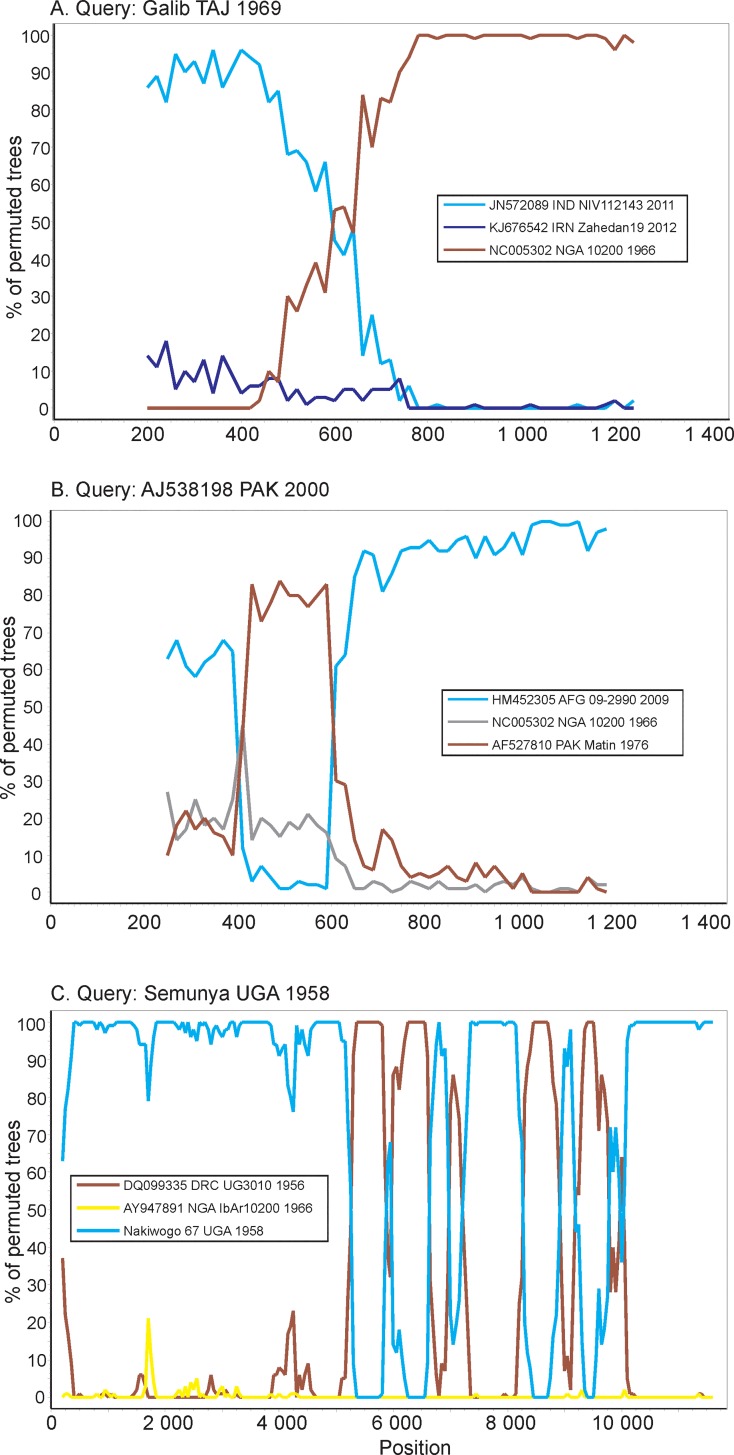
Bootscan analysis of exemplary CCHFV genomes. The Y axis shows the percentage of phylogenetic trees with a reliably supported grouping of a query sequence with sequences indicated in the legend in a sliding window that corresponds to the x axis. A. S segment, window 400 bp, step 20 bp. B. S segment, window 500 bp, step 20 bp. C. L segment, window 400 bp, step 50 bp.

Recombination within CCHFV genome segments was reported previously [17] and confirmed in subsequent studies [3]. CCHFV sequences were analyzed for recombination using the RDP 4.0 package, which utilizes a panel of algorithms. The detected cases were then visualized using bootscan graphs. Consistent with previous reports, several cases of significantly supported recombination events were found within the S segment (Fig 4A and 4B). In the L segment, only GtII (M-I) viruses isolated in DRC and Uganda displayed evidence of recombination. Interestingly, the pattern was highly mosaic and implied multiple exchanges within that group (Fig 4C). These recombination events were identified in the middle of individual chromatograms of the Nakiwogo strain sequenced in this work, ruling out experimental error (amplification of fragments of different genomes from a mixture of viruses). No reliably supported recombination events could be detected in the M segment; however, a few sequences had evidence of introduction of very short highly divergent sequences of unknown origin, suggestive of sequencing errors. In general, in CCHFV there were significantly fewer suggested recombination events than reassortment cases.

## Discussion

Phylogenetic analysis of all three genome segments suggested that CCHFV emerged in Africa only several thousand years ago. This value could be reproducibly obtained for different genome segments in this and other studies [9]. Dating of evolutionary events that occurred thousands of years ago can be questionable. While Bayesian phylogenetic inference is now the standard methodology, there have been concerns that dating of ancient nodes can be wrong by up to five orders of magnitude [24]. One of the main factors that could compromise ancestral node dating is the saturation of synonymous sites. For the poliovirus, it was estimated that mutation saturation could be achieved in approximately 100 years [25]. However, poliovirus has substitution rates of approximately 10^−2^ ssy, and CCHFV was estimated to have rates of approximately 10^−4^ ssy. Polioviruses of the same type may contain up to 25% nucleotide sequence differences in the VP1 genomic region commonly used for dating [26]. In the CCHFV sequences used here, the mean nucleotide sequence distance was 10%, and the maximum was 18%. Therefore, it is unlikely that mutation saturation had a critical impact on molecular dating. Nevertheless, until more incite is obtained in this field, the dates of ancestral nodes have to be treated with caution.

Virus introduction to Central and South Asia dated back to the Middle Ages. This was compatible with historical references describing a disease, which is now believed to have been CCHF, around 1100 in Middle Asia [27]. Then, CCHFV GtV was apparently introduced to Europe via the Volga Delta region only a few hundred years ago, and spread to the Balkans even more recently, supporting its classification as an emerging zoonosis. This relatively fast dynamics implies that CCHFV in Europe likely did not reach an ecological balance, and that the further spread of CCHFV is very likely. Unfortunately, no complete segment sequences were available for lineage VI (Europe 2), which was apparently introduced to Europe, probably via Turkey, also approximately 100 years ago [7]. It is surprising that the virus, which emerged in Africa several thousand years ago, was introduced and got established in Europe only so recently, given a proven capacity of migratory birds to carry ticks infected with African CCHFV variants [28] and the occasional detection of non-conventional CCHFV genotype variants in Europe [12, 29]. Moreover, it is surprising that only two virus introductions to Europe were successful, while numerous infected nymphs might have been transferred each year between continents by migratory birds. Currently there is no comprehensive data on the number of nymphs of competent tick species and the fraction of infected nymphs on migratory birds of different species. It is only known that many bird species are not efficient carriers of infected nymphs [28]. Further studies are necessary to understand the barriers to establishment of CCHF foci in nature.

Segment reassortment in CCHFV was first described over a decade ago [5] and has been confirmed in subsequent studies [3, 6, 22]. An increase of sequence samples over time provided evidence of more and more common reassortment. A recent study reported that 10 of 15 South African isolates were reassortant [22]. Another study used 30 complete genomes and identified 16 reassortment events [19]. Our analysis confirmed that reassortment is not a possibility, but a regular event, and the median half-life time of a non-recombinant virus was just 68–156 years in different segments. It is noteworthy that two of the non-reassortant nodes with the highest ages (257–834 years in different segments) included viruses of GtII (M I), which was represented by just three sequences. It is likely that the global sample was too small to detect reassortment within this group. Moreover, near-identical sequences were excluded from the analysis, thus pushing up the median half-life of a non-reassortant virus. Therefore, the suggested median rate of reassortment, which can be approximated to “once in 100 years”, may be regarded as a conservative estimate, and further studies may produce yet higher reassortment frequency estimates.

Due to reassortment, CCHFV genome segments generally had independent evolutionary patterns confined by ecological barriers to virus exchange (e.g., in the European GtV), but not by molecular limitations of segment compatibility, as reassortment between very distantly related viruses was observed. Natural reassortment is an established species criterion in the genera *Orthobunyavirus* and *Hantavirus* of the family *Bunyaviridae*, but not in the genus *Nairovirus* [30]. The frequent natural reassortment among CCHFV, but not with any other nairoviruses, implies that natural reassortment can also be considered as a species criterion in the genus *Nairovirus*.

Absence of segment reassortment between the European GtV and CCHFV from other regions contrasts with common gene transfer between Asian and African strains. It suggests that GtV could represent a spill-over from the main CCHFV gene pool, which then became reproductively isolated (not involved in gene transfer). This observation also supports the conclusion that CCHFV introduction to Europe was an exceptional event in the evolutionary history of the virus.

It is believed that RNA viruses existed on the eve of the cellular world [31]. The ecological niche occupied by ticks and small mammals have been likely present for a very long time. Therefore, it is surprising that CCHFV has such a recent evolutionary history. While dating of ancient evolutionary events may be arguable, the recent introduction of the virus to Europe is well supported by data. Therefore, arboviruses, deemed one of the most evolutionarily stable groups of RNA viruses because of strict constraints that accompany their capacity to replicate in two host systems [32], are an important potential source of emerging viruses.

## Supporting Information

S1 File KML file for visualization of S segment phylogeographic analysis. (KML)

S2 File KML file for visualization of M segment phylogeographic analysis. (KML)

S3 File KML file for visualization of L segment phylogeographic analysis. (KML)

S1 Table Oligonucleotides used to amplify CCHFV genome fragments. (DOCX)

S2 Table Coordinates of strain isolation locations. Coordinates were approximated to a country according to the Google country list table (https://developers.google.com/public-data/docs/canonical/countries_csv) or to a province/administrative region center for strains originating from Russia, China and Iran.
(XLSX)

## Abbreviations

CCHF(V): Crimean Congo Hemorrhagic Fever (virus)
Gt: genotype
MRCA: most recent common ancestor
ssy: substitutions/site/year
