# Supplementary material for: Phylogeography of Crimean Congo Hemorrhagic Fever Virus
Source: PLoS One. 2016 Nov 23;11(11):e0166744. doi: 10.1371/journal.pone.0166744 (PMC5120814; doi:10.1371/journal.pone.0166744)
Supplement: S1 Table — (DOCX) [file pone.0166744.s004.docx]

Table S1. Oligonucleotides used to amplify CCHFV genome fragments.

| Oligo name | Sequence |
| --- | --- |
| CCHF-L0001F | TCTCAAAGATATCAATCCCCC |
| CCHF-L0998F | ACTAAGGATTTGCTTGACCATCT |
| CCHF-L1121R | ATACAGTGCAGATGTCYAGTATCAT |
| CCHF-L1934F | ATAAGAAAYTCTCCAACRCACAG |
| CCHF-L2029R | CCAGCCRTARCAGTTKGCA |
| CCHF-L2952F | AGCAGGCAATGCGHAAAGG |
| CCHF-L3068R | TCCGCTCGAAYTCRTCAGTTAT |
| CCHF-L3963F | TGTTCAAGGAAGTRGCAGCWGA |
| CCHF-L4029R | CTATAATTCCTTCATAAGGTGTGCCAAT |
| CCHF-L4984F | AGAAAGGCATATGCTYTGGGA |
| CCHF-L5099R | TTGCTGCTTTCCTHACATTTGG |
| CCHF-L5519F | AGGTGACGGARACAACAAGYATAGT |
| CCHF-L6082F | TAAGGACTTTGCAAGCATHAATGA |
| CCHF-L6156R | CTTATGTCATATAGCTCTTCAGA |
| CCHF-L6399R | TCCTCTTTAGATCCACAGTCAAGTAT |
| CCHF-L7057F | CTGTCTTGCCAAYATGCGAAA |
| CCHF-L7148R | CACCTGAGATGCAGATRACTTT |
| CCHF-L8242R | TTCAGCTGTGGAACTTGYCCT |
| CCHF-L8976F | AGACAGGTGGTCYACCAAGCA |
| CCHF-L9080R | GAAAGGTGTCCAACCAGTGAGT |
| CCHF-L9999F | CTGTGAAGCGAGAYTCTGA |
| CCHF-L10123R | GTCTCCCCAGGTGYTGAACTT |
| CCHF-L10988F | ATTGAGGACATAGGCTTCAACAA |
| CCHF-L11059R | TTCCACAGGAGYTCACTYTTHAC |
| CCHF-L11790F | AGCAGTGTCAAGAGCAAATAAGC |
| CCHF-L12108R | TCTCAAAGAAATCGTTCCCCCC |
| CCHF-M0001F | TCTCAAAGAAATACTTGCGGCAC |
| CCHF-M0931F | CTGAAGGCCTGYTDGARTGGTG |
| CCHF-M1032R | TTCCCTCACCAGTCAWRAARAA |
| CCHF-M1888F | CAGGAGGGAAGGGGYCAYGT |
| CCHF-M2025R | CTCCAGTGCCYTTNGGTATCAT |
| CCHF-M2943F | GCTGTCTGCAAGCGYATGT |
| CCHF-M3077R | GCGGAGGTGCTRACNACACA |
| CCHF-M3951F | GAAGGTTTCTTTGACCTDATGCA |
| CCHF-M4155R | ACAGCCRTCCCADGACATCCA |
| CCHF-M4465F | TAGTTCTGGCATCTCHTGYAARGT |
| CCHF-M4599R | TTCCAAATTCAAGCTTYCTNGCCAT |
| CCHF-M5466R | TCTCAAAGATATAGTGGCGGCA |
| CCHF-S0001F | TCAAAGAAACACGTGCCGCT |
| CCHF-S0722F | TCAGGCCGTTCRGGRATAGC |
| CCHF-S0909R | GCAATGTGCTTRAGRAGGTTTGT |
| CCHF-S1692R | TCTCAAAGATATCGTTGCCGCA |
|  |  |
|  |  |
|  |  |

Oligo names include segment, 5’ end position (according to Genbank entries AY389361 for L, AF467768 for M and NC005302 for S segment, and orientation (Forward/Reverse).
